# Which Predictor, SctO2 or SstO2, Is more Sensitive for Postoperative Cognitive Dysfunction in Spine Surgery: A Prospective Observational Study?

**DOI:** 10.1111/os.13580

**Published:** 2022-11-16

**Authors:** Fei Guo, Shuaiying Jia, Qiyan Wang, Qinyu Liu, Mingquan Hu, Wenzhang Wang, Shijian Liu, Qiang Li, Bin Lu, Yeying Zheng

**Affiliations:** ^1^ Department of Anesthesiology Zigong Fourth People's Hospital Affiliated to Southwest Medical University Zigong China; ^2^ Department of Anesthesiology The Affiliated Hospital of North Sichuan Medical College Nanchong China; ^3^ Translational Medicine Center, the Second Affiliated Hospital Guangzhou Medical University Guangzhou China

**Keywords:** Cerebral tissue oxygen saturation, Hypertension, Postoperative cognitive dysfunction, Somatic tissue oxygen saturation, Spine surgery

## Abstract

**Objective:**

Patients undergoing spinal surgery in the prone position may experience venous stasis, often resulting in edema in dependent areas of the body, including the head, and increased postoperative cognitive dysfunction (POCD). Not only does POCD present challenges for post‐operative care and recovery, it can also cause permanent damage to the patient's brain and increase mortality and social costs. We aimed to clarify the incidence of POCD in patients with hypertension after prone spine surgery and to further determine the association between intraoperative somatic tissue oxygen saturation (SstO2)/cerebral tissue oxygen saturation (SctO2) and POCD.

**Methods:**

Patients with hypertension scheduled for open prone spine surgery from January 2020 to April 2021 were included in this single‐center, prospective, observational study. SctO2 and SstO2 were monitored by near‐infrared spectroscopy continuously throughout the surgery. The primary outcome was POCD assessed using the Mini‐Mental Status Examination (MMSE). The association of SstO2 and SctO2 with POCD was evaluated with unadjusted analyses and multivariable logistic regression.

**Results:**

One hundred and one of 112 identified patients were included, 28 (27.8%) of whom developed POCD. None of the investigated SctO2 indices were predictive of POCD. However, the patients with POCD had greater decreases in intraoperative absolute SstO2 and relative SstO2 than the patients without POCD (*P* = 0.037, *P* = 0.036). Moreover, three SstO2 indices were associated with POCD, including a greater absolute SstO2 decrease (*P* = 0.021), a greater relative SstO2 decrease (*P* = 0.032), and a drop below 90% of the baseline SstO2 (*P* = 0.002), independent of ASA III status, preoperative platelets and postoperative sepsis. In addition, there was no correlation between intraoperative SctO2 and intraoperative SstO2 or between their respective absolute declines.

**Conclusion:**

Twenty‐eight (27.7%) of 101 patients developed POCD in patients with hypertension undergoing prone spine surgery, and intraoperative SstO2 is associated with POCD, whereas SctO2 shows no association with POCD. This study may initially provide a valuable new approach to the prevention of POCD in this population.

## Graphical abstract

The patients with postoperative cognitive dysfunction (POCD) had a higher decrease in intraoperative absolute somatic tissue oxygen saturation (SstO2) decrease (4.9 ± 3.8 *vs* 3.6 ± 2.6, *P* = 0.037) (A) and a higher decrease in intraoperative relative SstO2 decrease (7.4 ± 5.6 *vs* 5.3 ± 3.8, *P* = 0.036) (B). The proportion below 90% of baseline SstO2 in the patients with POCD were significantly higher than patients without (32.1% *vs* 5.5%, *P* < 0.001) (C). A negative correlation between the parameters of cerebral tissue oxygen saturation (SctO2) (e.g., the absolute decrease) and the length of postoperative hospital stay (r^2^ = 0.082, *P* = 0.004) (D).

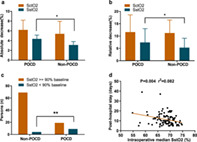

## Introduction

Patients undergoing open‐prone spine surgery may lead to venous stasis, which often results in edema in dependent body parts, including the head, leading to increased postoperative cognitive dysfunction (POCD).^1^ POCD has been associated with a prolonged hospital stay, long‐term neurocognitive deterioration, increased mortality, and increased social costs.^2^, ^3^, ^4^ Studies have confirmed that various strategies such as electroacupuncture,^5^ perioperative ulinastatin combined with dexmedetomidine,^6^ and preoperative dexamethasone^7^ can reduce the incidence and severity of POCD. Many animal and clinical studies have focused on the role of CNS inflammation in the development and progression of POCD, but the specific mechanisms need to be elucidated,^8^ and no anesthetic or surgical strategy to date has been proven to eliminate the cognitive impact.^9^ Identifying predictors for the development of POCD is therefore vital for early prevention and treatment of this condition.^4^, ^10^

Intraoperative hypoxia and hypoperfusion have frequently been regarded as one of the important factors contributing to the development of POCD.^1^, ^11^ Near‐infrared spectroscopy (NIRS) can noninvasively measure regional cerebral oxygen saturation (SctO2) and/or somatic tissue oxygen saturation (SstO2) at the microvascular level, thereby enabling the detection of mismatches between oxygen supply and consumption.^12^, ^13^, ^14^ In recent years, a large number of studies have reported associations between NIRS measurements (SctO2 and/or SstO2) and neurological complications.^15^, ^16^, ^17^, ^18^, ^19^, ^20^ However, these results are mainly from supine surgery and may not apply to prone surgery. In prone surgery, SctO2 is likely to be affected by edema of the monitoring site.^21^ What is more, it is reported that there are a large discrepancy and inconsistent correlation between intraoperative SctO2 and SstO2 measurements in prone spine surgery, suggesting their non‐interchangeability.^22^ In clinical practice, it is very vital to clarify which NIRS measurement (intraoperative SctO2 or intraoperative SstO2) is a better association with POCD in prone surgery, but few studies to date have been performed.^15^ A recent study of prone spine surgery showed a good association between postoperative complications and SstO2 but almost no association with SctO2.^21^ Thus, we hypothesized that in a prone position, SctO2 is affected by edema and venous‐return disorders at the monitoring position, but SstO2 is not.

What is more, hypertension can increase the brain's threshold for self‐regulation of blood pressure, cause blood–brain barrier breakdown, and frequently result in labile hemodynamics during surgery, which is more likely to lead to cognitive decline.^1^, ^23^, ^24^ However, the incidence of POCD after open‐prone spine surgery in patients with hypertension is not known. Therefore, this prospective observational study aimed to clarify the incidence of POCD in patients with hypertension after open prone spine surgery, the relationship between SctO2 and SstO2, the relationship between SctO2/SstO2 and POCD, and the relationship between SctO2/SstO2 and hospital stay.

## Methods

### Study Design

This prospective observational study was registered as a clinical trial registration number ChiCTR1900028392 on 20 December 2019. The inclusion criteria were as follows: (i) patients with hypertension; (ii) patients undergoing elective open posterior lumbar or/and thoracic spine surgery in a prone position; and (iii) age > 18 years. The exclusion criteria were as follows: (i) patient refusal; (ii) American Society of Anesthesiologists (ASA) physical status score > III; (iii) other operations; (iv) skin conditions affecting oximetry probe measurements; (v) a preoperative Mini‐Mental State Examination (MMSE) score < 18; and (vi) literacy problems, language difficulties, and hearing or visual impairment.

The study protocol was approved by the Institutional Committee for Medical Ethics of Zigong Fourth People's Hospital Affiliated with Southwest Medical University, Zigong, Sichuan, China (009/2019). All methods were carried out in compliance with the Declaration of Helsinki and written informed consent was obtained from each participant before surgery. This current manuscript adheres to the applicable STROBE guidelines (http://www.equator-network.org/).

### Anesthesia

No premedication was given. After the patients entered the operating room, radial arterial cannulation, arterial blood gas analysis, and bispectral index (BIS) monitoring were initiated with the patients breathing room air. General anesthesia was induced using midazolam (0.05 mg/kg), sufentanil (0.3–0.4 μg/kg), cisatracurium (0.2 mg/kg) and etomidate (0.2 mg/kg). Then, propofol (0–1.5 mg/kg) was used according to the BIS before tracheal intubation was performed. Under the direction of the same attending anesthesiologist, general anesthesia was maintained at BIS values from 40% to 60% using sevoflurane in an oxygen‐air mixture (oxygen concentration 40%). The respiratory rate was set to maintain an end‐tidal CO2 of 35–40 mmHg. Sufentanil and cisatracurium were added intermittently by the attending anesthesiologist according to surgical needs. Ephedrine was usually used to treat hypotension (systolic blood pressure < 90 mmHg or > 30% decrease from baseline), and when ephedrine had an insufficient effect, deoxyepinephrine was used. Tranexamic acid was routinely administered 30 minutes before surgical skin cutting. Cell salvaging was available for all patients. After surgery, analgesia through incision infiltration was routinely performed, and oral analgesic drugs and/or self‐controlled electronic analgesia pumps were utilized as needed.

### Tissue Oxygenation Monitoring

Tissue oxygenation was monitored using a tissue oximeter (EGOS‐600A, Eqin tissue oxygen saturation monitor, Suzhou, China) based on NIRS. The oximeter had four cables, with each cable connected to a NIRS sensor. Two NIRS sensors were placed on the upper forehead, and one finger above the eyebrow, to monitor the left and right frontal lobe SctO2. The other two NIRS sensors were placed on the waist in the midaxillary line, two fingers below the costal margin, to monitor the SstO2 at the left and right sides of the waist. All four NIRS sensors were placed on the patient upon entry into the operating room. The patient's baseline SctO2 and SstO2 were acquired before anesthetic induction while the patient breathed room air. Tissue oxygenation monitoring and data recording were started when anesthetic induction was initiated and were discontinued when the operation was completed. The oximeter was placed at the back of the anesthesia machine and concealed from the anesthesiologists.

The tissue oximeter generated a new data point every 2 s, and the data were continuously recorded by a computer. The SctO2/SstO2 data points were used for tissue oxygenation index derivation. Eight indices of tissue oxygenation were derived, including the baseline, minimum, mean, and median values, the absolute SctO2 decrease, the relative SctO2 decrease, the area under the curve (AUC), and the number of desaturation episodes to <80% or < 90% of the baseline value. The AUC was the sum of the differences between a specific threshold and the data point of every minute that was below the threshold. For each index, SctO2 and SstO2 were treated independently.

### Clinical Covariates

Patient characteristics were collected, including age, sex, weight, height, current smoking status, alcohol consumption, and ASA. Past medical histories, including diagnoses of hypertension, coronary artery disease, previous stroke, diabetes mellitus, chronic obstructive pulmonary disease, chronic hepatitis B, and chronic kidney disease were recorded. Surgical information included fusion (yes or no), the number of operating segments, estimated blood loss, and surgical time. The use of ephedrine and phenylephrine, fluid infusion volume, lactate during suturing, urine output, and blood transfusion were recorded. After surgery, the time of tracheal extubation, length of PACU stay, numerical pain score (NPS, at 1 days and 3 days postoperatively), and 24‐h postoperative drainage volume were also recorded.

### Postoperative Outcome Measure

The primary outcome measure was POCD. The testing protocol was the quick assessment scale of the MMSE, which is widely used as a screening tool for cognitive dysfunction.^25^ The MMSE addresses orientation to time and place, registration, attention, calculations, recall, repetition, and complex commands^25^ and is known to be less sensitive when applied repeatedly.^26^ Therefore, in this study, cognitive function tests were performed only on Day 1 before surgery and on Day 4 after surgery. When administering the MMSE a second time, the order of test elements was varied to increase the sensitivity of the test.

Cognitive function was tested by a special clinician with training in cognitive assessments. The testing clinician was blinded to each patient's surgical and anesthetic conditions. In this study, a decrease of 2 points or more from the preoperative score was considered indicative of a significant decline in cognitive function and defined as POCD.^15^

Primary postoperative complications were identified based on chart reviews of the electronic medical records at the Zigong Fourth People's Hospital Affiliated with Southwest Medical University. Information on the length of postoperative hospital stay and total medical costs was also recorded.

### Statistical Analysis

Statistical analysis was performed using IBM SPSS version 23 (IBM, Chicago, IL). A two‐sided *P* value < 0.05 was considered statistically significant. The Kolmogorov–Smirnov test was used to determine whether continuous data conformed to a normal distribution. Normally distributed data are presented as the mean (±standard deviation), and data with a skewed distribution are presented as the median with the first and third quartiles. Categorical variables are expressed as n (%). Continuous data were compared by the independent *t*‐test or Mann–Whitney U‐test, and categorical data were compared between groups with and without POCD using the chi‐square test or Fisher's exact test. Repeated measurements between the two groups were compared by the paired *t*‐test. Correlations between two continuous variables were analyzed using Spearman or Pearson correlation analysis.

Baseline patient characteristics and perioperative characteristics that were significant (at a threshold *P* < 0.2) in the univariable logistic regression analysis were entered into a forward multivariable logistic regression model. The multicollinearity test was performed on all included variables in advance, and variables with a variance inflation factor >5 were excluded. The goodness of fit of the final multivariable logistic regression model was evaluated using the Hosmer–Lemeshow test. The predictive power of the final model was assessed by a receiver operating characteristic (ROC) curve, and the AUC was calculated to evaluate POCD prediction performance. Finally, to clarify the relationship between SctO2/SstO2 parameters and POCD and avoid the influence of collinearity among parameters, the SctO2/SstO2 parameters (*P* < 0.2 in the univariable logistic regression analysis) were adjusted one by one using independent risk factors obtained in the final multivariate logistic regression model.

Using PASS 15 software (NCSS Statistical Software) and assuming that the odds ratio for POCD among patients with cerebral/somatic desaturation defined by <90% baseline was 5.82 relative to patients without cerebral/somatic desaturation defined by <90% baseline,^19^ we would require a minimum sample of 87 patients that received NIRS measurement when the incidence of POCD was 20%, and the incidence of cerebral/somatic desaturation defined by <90% baseline was 30%, in order to achieve a Type I error of 0.05 (two tails) and a power function of 0.95. In addition, assuming a 15% dropout rate, the sample size was inflated from 87 to 100 patients.

## Results

### Study Population

**Fig. 1:**
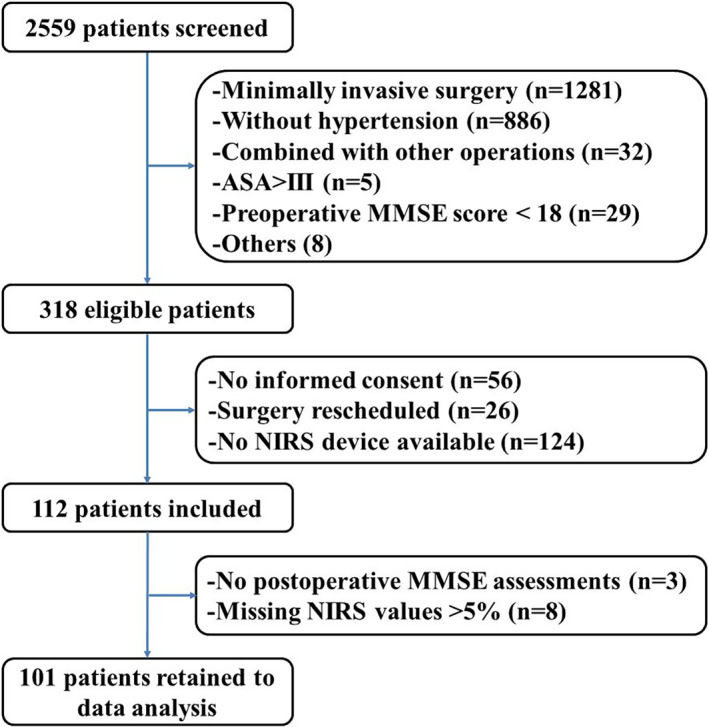
Flow diagram presenting patient enrolment. ASA, American Society of Anesthesiologists; MMSE, Mini‐Mental State Examination; NIRS, near‐infrared spectroscopy

**Fig. 2:**
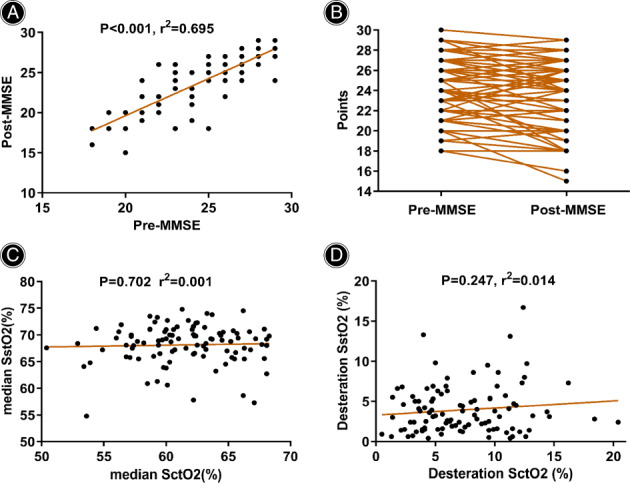
Characteristics of MMSE and SctO2/SstO2 parameters in 101 patients. The postoperative MMSE was positively correlated with preoperative MMSE (A) and presented a downward trend (B). There was no correlation between intraoperative SctO2 and intraoperative SstO2 (C) or between their respective absolute declines (D)

From January 2020 until April 2021, 112 patients scheduled for elective posterior lumbar or/and thoracic spine open surgery were included in this prospective observational study (Fig. 1). Eleven patients were excluded from the data analysis; three patients had no postoperative MMSE assessments, and eight patients had more than 5% missing SctO2/SstO2 values. Ultimately, 101 patients were included in the data analysis. The postoperative MMSE score was positively correlated with the preoperative MMSE score (r^2^ = 0.695, *P* < 0.001, Fig. 2A), while the postoperative MMSE score decreased compared with the preoperative MMSE score (24.9 ± 2.9 *vs* 24.1 ± 3.3, *P* < 0.001, Table 1 and Fig. 2B). No correlation was found between intraoperative SctO2 and intraoperative SstO2 (*P* = 0.702, Fig. 2C) or between their respective absolute declines (*P* = 0.247, Fig. 2D). Of the 101 patients, 28 (27.7%) developed POCD. A total of 1, 2, 3 and 4 segments were surgically treated in 62 (61.4%), 34 (33.7%), 4 (4.0%) and 1 (1.0%) of the patients, respectively. Decompression and fusion were performed in 75 (74.3%) and 74 (73.3%) patients, respectively.

**TABLE 1:** Perioperative characteristics in the study population, and comparison between the two groups

Variables	All patients (n = 101)	POCD (n = 28)	No‐POCD (n = 73)	Test statistic	*P*‐values
Patient characteristics					
Age (yr)	64.8 ± 9.3	67.8 ± 8.9	63.7 ± 9.3	2.026	0.045
Male sex, n (%)	42 (41.6)	10 (35.7)	32 (43.8)	0.549	0.459
BMI (kg.m^−2^)	24.7 ± 3.2	24.8 ± 3.2	24.6 ± 3.2	0.179	0.858
ASA II, n (%)	54 (53.5)	9 (32.1)	45 (61.6)	7.079	0.008
ASA III, n (%)	47 (46.5)	19 (67.9)	28 (38.4)	7.079	0.008
Cognitive status					
Education (years)	6 (4,11)	6 (4,11)	6 (4,11)	0.223	0.818
Preoperative MMSE	24.9 ± 2.9	24.6 ± 3.2	24.9 ± 2.9	0.460	0.647
Postoperative MMSE	24.1 ± 3.3	21.8 ± 3.6	25.0 ± 2.7	5.005	<0.001
Past medical history, n (%)					
Hypertension				1.017	0.601
grade 1	40 (39.6)	9 (22.5)	31 (77.5)		
grade 2	34 (33.7)	10 (29.4)	24 (70.6)		
grade 3	27 (26.7)	9 (33.3)	18 (66.7)		
Coronary artery disease	4 (4.0)	1 (3.6)	3 (4.1)	‐	1.000
Previous stroke	2 (2.0)	0	2 (2.7)	‐	1.000
Diabetes mellitus	23 (22.8)	6 (21.4)	17 (23.3)	0.040	0.842
COPD	3 (3.0)	1 (3.6)	2 (2.7)	‐	1.000
Chronic hepatitis B	7 (6.9)	3 (10.7)	4 (5.5)	‐	0.393
Chronic kidney disease	9 (8.9)	5 (17.9)	4 (5.5)	‐	0.111
Preoperative Laboratory tests					
Hemoglobin (g/L)	132 ± 15	132 ± 14	133 ± 16	0.272	0.786
White blood cell (10^9^/L)	6.5 ± 1.9	6.6 ± 1.7	6.5 ± 2.0	0.201	0.841
Platelet (10^9^/L)	167 ± 56	194 ± 68	157 ± 47	3.110	0.002
INR	1.05 ± 0.07	1.04 ± 0.09	1.05 ± 0.06	0.627	0.532
Alanine transaminase (U)	21.2 (16.8, 29.6)	20.6 (15.4, 37.4)	22.0 (16.8, 28.2)	0.125	0.900
Aspartate aminotransferase (U)	23.1 (18.2, 29.4)	24.3 (17.8, 29.2)	22.8 (18.4, 29.7)	0.121	0.903
Albumin (g/L)	41.0 ± 3.7	40.5 ± 3.9	41.2 ± 3.7	0.768	0.444
Glomerular filtration rate (ml/min)	99 (84,121)	99 (85,115)	99 (82,126)	0.679	0.497
Surgical information					
Number of segments (n)				1.088	0.843
1	62 (61.4)	19 (67.9)	43 (58.9)		
2	34 (33.7)	8 (28.6)	26 (35.6)		
3	4 (4.0)	1 (3.6)	3 (4.1)		
4	1 (1.0)	0	1 (1.4)		
Decompression, n (%)	75 (74.3)	24 (85.7)	51 (69.9)	2.660	0.103
Fusion, n (%)	74 (73.3)	23 (82.1)	51 (69.9)	1.558	0.212
Surgical time (minutes)	165 (140, 197)	173 (141, 201)	164 (137, 195)	0.732	0.464
Intraoperative management					
Fluid infusion volume (ml)	1700 (1300, 2000)	1650 (1250, 2150)	1700 (1300, 2000)	0.042	0.967
Transfusion, n (%)	2 (2.0)	2 (7.1)	0	‐	0.075
Estimated blood loss (ml)	300 (200, 500)	325 (200, 500)	300 (200, 525)	0.732	0.464
Urine output (ml)^a^	250 (150, 400)	200 (135, 450)	250 (200, 300)	0.433	0.665
Lactate when suturing (mmol/L)	1.39 (1.08, 1.75)	1.42 (1.15, 1.79)	1.39 (1.07, 1.74)	0.330	0.741
Ephedrine, n (%)	82 (81.2)	21 (75.0)	61 (83.6)	0.971	0.324
Phenylephrine, n (%)	21 (20.8)	10 (35.7)	11 (15.1)	5.238	0.022
Postoperative management					
Extubation time (minutes)	21 (15,33)	21 (15,34)	23 (15,33)	0.304	0.761
Length of PACU stay minutes)	50 (40,67)	51 (40,67)	50 (40,67)	0.300	0.764
NPS in postoperative 1d (points)	1 (1,2)	1 (1,2)	1 (1,2)	0.919	0.358
NPS in postoperative 3d (points)	1 (1,1)	1 (1,2)	1 (1,1)	1.526	0.127
24‐h postoperative drainage (ml)	150 (53,227)	155 (80,298)	100 (23,205)	2.012	0.044
Postoperative complications					
Cerebrospinal leak, n (%)	8 (7.9)	5 (17.9)	3 (4.1)	‐	0.036
Postoperative sepsis, n (%)	9 (8.9)	5 (17.9)	4 (5.5)	‐	0.111
Thrombocytopenia (Platelet<100,000), n (%)	16 (15.8)	5 (17.9)	11 (15.1)	‐	0.765

Abbreviations: ASA, American Society of Anesthesiologists; BMI, body mass index; COPD, chronic obstructive pulmonary disease; INR, international normalized ratio; MMSE: Mini‐Mental State Examination; NPS, numerical pain score; PACU, postanesthesia care unit.

^a^ Only 64 patients were included because the remaining 47 patients did not receive intraoperative catheterization due to ERAS implementation.

### Characteristics between the Two Groups

The patients with POCD were older, had higher rates of chronic kidney disease and ASA III, and had higher platelet counts than the patients without POCD (Table 1). Education levels and preoperative MMSE scores were similar in both groups (*P* = 0.818 and *P* = 0.647, respectively), while the patients with POCD scored lower than those without POCD on the postoperative MMSE (*P* < 0.001). In perioperative management and postoperative complications, POCD patients had a higher proportion of intraoperative phenylephrine use, more 24‐h postoperative drainage, and a higher proportion of postoperative cerebrospinal leak. However, no difference in intraoperative blood loss or the proportion of intraoperative ephedrine use was noted between the two groups.

### Comparison of SctO2 and SstO2 between the Two Groups

The univariable comparison of intraoperative NIRS (both SctO2 and SstO2) parameters between the patients with and without POCD is shown in Table 3. The baseline SctO2, lowest intraoperative SctO2, mean SctO2, and median SctO2 were similar between the patients with and without POCD (*P* = 0.593, 0.947, 0.728, and 0.722, respectively). Likewise, the intraoperative absolute SctO2 decrease, the relative SctO2 decrease, the AUCs of intraoperative SctO2 values below 60%, 55%, and 50%, the number of desaturation episodes to below 80% of the baseline SctO2 and the number of desaturation episodes to below 90% of the baseline SctO2 did not differ between the groups (*P* = 0.649, 0.730, 0.296, 0.239, 0.201, 0.682, and 0.561, respectively). Generally, none of the investigated SctO2 variables were predictive of POCD.

**Fig. 3:**
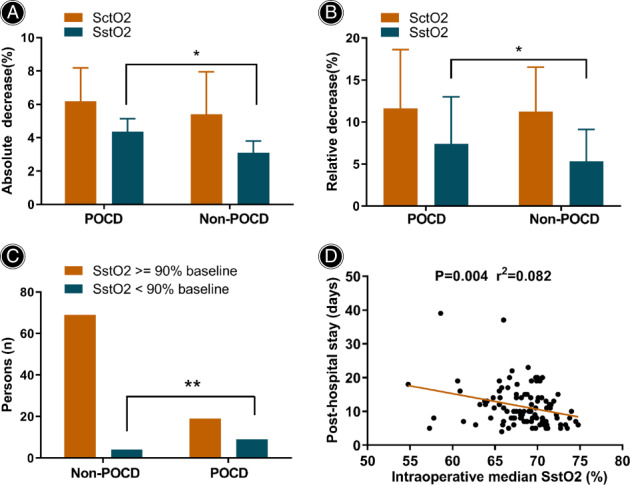
The comparison between SstO2/SctO2 and POCD, and the association between SstO2 and postoperative hospital stay. The patients with POCD had a higher decrease in intraoperative absolute SstO2 decrease (4.9 ± 3.8 *vs* 3.6 ± 2.6, *p* = 0.037) (A) and a higher decrease in intraoperative relative SstO2 decrease (7.4 ± 5.6 *vs* 5.3 ± 3.8, *P* = 0.036) (B). The proportion below 90% of baseline SstO2 in patients with POCD was significantly higher than in patients without (32.1% *vs* 5.5%, *P* < 0.001) (C). A negative correlation between the parameters of SctO2 (e.g., the absolute decrease) and the length of postoperative hospital stay (*r* ^2^ = 0.082, *P* = 0.004) (D)

In contrast to SctO2 measured at the forehead, the patients with POCD had a greater intraoperative absolute SstO2 decrease and relative SstO2 decrease than the patients without POCD (4.9% ± 3.8% *vs* 3.6% ± 2.6%, *P* = 0.037; 7.4% ± 5.6% *vs* 5.3% ± 3.8%, *P* = 0.036, respectively; Table 2 and Fig. 3A,B). The proportion of desaturation episodes to below 90% of the baseline SstO2 in the patients with POCD was significantly higher than that in patients without POCD (32.1% *vs* 5.5%, *P* < 0.001, Fig. 3C), but 19 (67.9%) of the 28 patients who developed POCD did not experience intraoperative SstO2 desaturation episodes to below 90% of the baseline value. Unfortunately, the number of desaturation episodes to below 80% of the baseline SstO2 did not differ between the two groups, as the frequency of such episodes to below 80% of the baseline SctO2 was only two, with one case in each group. In addition, other investigated SstO2 variables, including the baseline SstO2, lowest intraoperative SstO2, mean SstO2, and median SstO2 did not differ significantly between the groups.

**TABLE 2:** Intraoperative cerebral and somatic tissue oxygenation in the study population, and comparison between the two groups

Variables	All patients	POCD(n = 28)	No‐POCD(n = 73)	Test statistic	*P*‐values
SctO2					
Baseline SctO2 (%)	64.2 ± 3.9	64.6 ± 4.8	64.1 ± 3.5	0.536	0.593
Lowest SctO2 (%)	56.9 ± 3.9	56.9 ± 4.8	56.9 ± 3.6	0.066	0.947
SctO2 mean (%)	62.1 ± 3.7	62.3 ± 4.3	62.0 ± 3.5	0.349	0.728
SctO2 median (%)	61.5 ± 3.9	61.7 ± 4.4	61.4 ± 3.8	0.356	0.722
Absolute SctO2 decrease (%)	7.4 ± 4.0	7.6 ± 4.8	7.2 ± 3.6	0.457	0.649
Relative SctO2 decrease (%)	11.3 ± 5.8	11.6 ± 7.0	11.2 ± 5.3	0.346	0.730
SctO2 AUC 60% (%*min)	17 (0,235)	5 (0,212)	27 (0,226)	1.045	0.296
SctO2 AUC 55% (%*min)	0 (0,0)	0 (0,2)	0 (0,0)	1.177	0.239
SctO2 AUC 50% (%*min)	0 (0,0)	0 (0,0)	0 (0,0)	1.278	0.201
Desaturation <80% of baseline, n (%)	8 (7.9)	3 (10.7)	5 (6.8)	‐	0.682
Desaturation <90% of baseline, n (%)	53 (52.5)	16 (57.1)	37 (50.7)	0.338	0.561
SstO2					
Baseline SstO2 (%)	68.3 (64.9,70.3)	67.8 (64.6,70.0)	68.4 (64.9,70.4)	0.410	0.682
Lowest SstO2 (%)	64.0 (61.0,66.4)	62.6 (60.6,65.2)	64.5 (61.4,66.5)	1.707	0.088
SstO2 mean (%)	68.7 (66.2,70.5)	68.0 (65.8,70.0)	68.7 (67.1,70.6)	1.267	0.205
SstO2 median (%)	68.8 (66.2,70.6)	67.8 (65.7,69.8)	68.9 (67.2,70.6)	1.354	0.176
Absolute SstO2 decrease (%)	3.9 ± 3.0	4.9 ± 3.8	3.6 ± 2.6	2.120	0.037
Relative SstO2 decrease (%)	5.9 ± 4.4	7.4 ± 5.6	5.3 ± 3.8	2.128	0.036
SstO2 AUC 60% (%*min)	0 (0,0)	0 (0,0)	0 (0,0)	0.083	0.933
SstO2 AUC 55% (%*min)	0 (0,0)	0 (0,0)	0 (0,0)	1.182	0.237
SstO2 AUC 50% (%*min)	‐	‐	‐	‐	‐
Desaturation <80% of baseline, n (%)	2 (2.0)	1 (3.6)	1 (1.4)	‐	0.480
Desaturation <90% of baseline, n (%)	13 (12.9)	9 (32.1)	4 (5.5)	‐	0.001

Abbreviations: AUC, area under the curve; SctO2, Cerebral tissue oxygen saturation; SstO2, Somatic tissue oxygen saturation.

### Associations between SctO2/SstO2 and POCD

The suspicious risk factors identified for POCD were the variables with *P*< 0.2 in the univariate logistic regression, which can be found in Supplementary Table S1 online, including age, ASA III, education level, preoperative chronic kidney disease, preoperative platelet count, intraoperative decompression, intraoperative phenylephrine, postoperative cerebrospinal leak, 24‐h postoperative drainage volume, postoperative sepsis and numerical pain score at postoperative Day 3. Finally, three SstO2 parameters were associated with POCD, including a greater absolute SstO2 decrease (odds ratio [OR], 1.223; 95% confidence interval [CI], 1.031–1.451; *P* = 0.021), a greater relative SstO2 decrease (OR, 1.138; 95% CI, 1.011–1.281; *P* = 0.032) and a drop below 90% of the baseline SstO2 (OR, 11.388; 95% CI, 2.367–54.785; *P* = 0.002), independent of ASA III status, preoperative platelet counts and postoperative sepsis (Table 3).

**TABLE 3:** Univariable and multivariable logistic regression analysis of the SctO2/SstO2 parameters for prediction of POCD

Variables	Univariable			Multivariable		
	OR	95% CI	P‐values	OR	95% CI	*P*‐values
ASA III (*vs* ASA II)	3.393	(1.348, 8.538)	0.009	3.992	(1.434, 11.112)	0.008
Platelet, per 10^9^/L	1.012	(1.004, 1.021)	0.005	1.012	(1.003, 1.021)	0.009
Postoperative sepsis	3.750	(0.928, 15.160)	0.064	5.201	(1.057, 25.583)	0.042
Cerebral tissue oxygen saturation (SctO2)						
Baseline SctO2, per %	1.032	(0.921, 1.155)	0.589	‐	‐	‐
Lowest SctO2, per %	1.004	(0.898, 1.122)	0.947	‐	‐	‐
Absolute SctO2 decrease, per %	1.026	(0.920, 1.145)	0.645	‐	‐	‐
Relative SctO2 decrease, per %	1.014	(0.940, 1.093)	0.727	‐	‐	‐
SctO2 mean, per %	1.021	(0.908,1.150)	0.725	‐	‐	‐
SctO2 median, per %	1.021	(0.913, 1.142)	0.719	‐	‐	‐
SctO2 AUC 60%, per %*min	1.000	(0.999, 1.001)	0.624	‐	‐	‐
SctO2 AUC 55%, per %*min	1.002	(0.998, 1.005)	0.314	‐	‐	‐
SctO2 AUC 50%, per %*min	0.994	(0.961, 1.029)	0.747	‐	‐	‐
Desaturation <80% of baseline	1.632	(0.363, 7.336)	0.523	‐	‐	‐
Desaturation <90% of baseline	1.297	(0.539,3.121)	0.561	‐	‐	‐
Somatic tissue oxygen saturation (SctO2)						
Baseline SctO2, per %	0.992	(0.891, 1.105)	0.883	‐	‐	‐
SctO2 minimum, per %	0.939	(0.859,1.026)	0.162	0.969	(0.871, 1.077)	0.558
SctO2 mean, per %	0.921	(0.818, 1.037)	0.174	0.934	(0.811,1.076)	0.346
SctO2 median, per %	0.919	(0.818,1.032)	0.153	0.922	(0.802, 1.060)	0.256
Absolute SctO2 decrease, per %	1.159	(1.003,1.339)	0.046	1.223	(1.031, 1.451)	0.021
Relative SctO2 decrease, per %	1.105	(1.003, 1.218)	0.044	1.138	(1.011, 1.281)	0.032
SctO2 AUC60, per %*min	1.002	(0.999, 1.006)	0.166	1.003	(0.998, 1.007)	0.260
SctO2 AUC55, per %*min	1.040	(0.917, 1.179)	0.546	‐	‐	‐
SctO2 AUC50, per %*min	‐	‐	‐	‐	‐	‐
Desaturation <80% of baseline	2.667	(0.161,44.155)	0.493	‐	‐	‐
Desaturation <90% of baseline	8.171	(2.266, 29.465)	0.001	11.388	(2.367, 54.785)	0.002

Abbreviations: ASA, American Society of Anesthesiologists; AUC, area under the curve; CI, confidence interval; OR, odds ratio.

### Associations between SctO2/SstO2 and Postoperative Hospital Stay

There was no statistical difference in the length of postoperative hospital stay between the two groups (*P* = 0.367, Table 2). However, in the 101 patients, negative correlations were identified between the SstO2 parameters (e.g., the absolute decrease) and the length of postoperative hospital stay (*r* ^2^ = 0.082, *P* = 0.004, Fig. 3D), while the SctO2 parameters showed no such correlations.

## Discussion

### Main Findings of the Study

This prospective observational study found that 28 (27.7%) of 101 patients with hypertension undergoing open posterior spine surgery developed POCD. The patients with POCD had a greater intraoperative absolute SstO2 decrease and relative SstO2 decrease than the patients without POCD, and the proportion of desaturation episodes to below 90% of the baseline SstO2 in the patients with POCD was significantly higher than that in patients without POCD. Moreover, POCD was strongly associated with the decrease in intraoperative SstO2 but showed no correlation with SctO2.

### No Correlation between SctO2 and SstO2

Of great interest is the lack of correlation between intraoperative SctO2 and SstO2. SctO2 is often used to avoid cerebral oxygen deficiency and prevent cognitive disorder,^17^ and SstO2 is also increasingly used to avoid peripheral oxygen deficiency and prevent postoperative complications.^27^ In the setting of open posterior spine surgery, few studies have monitored SctO2 and SstO2 simultaneously. The results of this study showed that both SctO2 and SstO2 were non‐invasive tools for assessing tissue oxygenation adequacy, whereas there was no correlation between intraoperative SctO2 and SstO2 or between their respective declines. These results may suggest that the correlations between the tissue oxygen saturation at different sites and POCD may be significantly different. The strategy of multisite monitoring may be beneficial at present, and identifying the best monitoring site is important to further improve patient outcomes.^22^

### No Correlation between SctO2 and POCD

In this study, no correlation was found between any indicator of intraoperative SctO2 in the forehead and POCD, which is similar to the result of a previous study that found no correlation between intraoperative SctO2 and postoperative complications after spine surgery.^21^ However, this result was inconsistent with those of other studies showing a good correlation between SctO2 and POCD.^28^ Although many preventive measures were implemented, the nature of the prone position may have still affected SctO2 measurements in this study for two reasons. First, all patients were in the prone position, which might result in cephalic and facial venous pooling. Second, patients in the prone position often develop edema in the downward part of the body, including the forehead.^21^ Therefore, the tissue oxygen saturation during surgery, monitoring in the downward part of the body including the forehead, may not be sensitive to enable detection of mismatches between oxygen supply and consumption. Unfortunately, recent findings in prone surgery are conflicting, and postural factors may not be able to explain all causes of the differences in the SctO2 monitoring results.^15^, ^21^

### SstO2 Was Associated with POCD

In contrast, the present study found that intraoperative SstO2 was significantly associated with POCD during spine surgery. Thus, we verified the previous hypothesis that SctO2, but not SstO2, measured at the forehead is affected by prone positioning, which also implied a potentially modifiable causal link, indicating that persistent somatic tissue desaturation should be avoided. This result is similar to that of Meng *et al*.^21^ who found that muscular tissue desaturation was significantly associated with postoperative complications. However, in this study, the monitoring site was the midaxillary line of the waist at the same height as the operation, while in their study, the lower leg was monitored. The main reasons why we did not choose the lower leg or the more common arm sites (especially the thenar muscle) for SstO2 measurement are as follows: first, the bilateral thenar muscles were usually used for evoked potential monitoring in our patients. Second, the monitoring points that we selected were almost at the same level as the vital organs and the brain, where hemodynamics might be more similarly affected by relative positioning. Third, the sites we monitored may include muscle tissue and visceral organs that are sensitive to ischemia and hypoxia, such as the small intestine, colon, and kidney. Nearly all internal organs are less capable of self‐regulation than the brain and heart.^29^ Intuitively, during periods of hemodynamic instability, the lowest‐priority sites may have better correlations with postoperative complications. Therefore, our results confirmed that intraoperative SstO2 but not SctO2 was associated with POCD during spine surgery.

### The Prevention and Treatment for POCD

The pathogenesis of POCD may be mainly affected by surgical trauma, anesthesia and the health status of patients; effective treatment of depression before surgery, no anticholinergic drugs before surgery, reduction of surgical trauma, prevention of postoperative infection and reduction of postoperative pain are beneficial to reduce POCD.^30^ For example, Sciard *et al*. have shown that good perioperative pain management and orthopedic surgical techniques (especially when surgical options are short and without bone cement) can decrease the incidence of POCD by reducing the patient's inflammatory response.^31^ In cardiac surgery, minimally invasive procedures may reduce the incidence of POCD, and prolonged mild hypothermia and slow rewarming may prevent the development of cognitive impairment after cardiac surgery.^32^ What is more, many preventive measures are also effective. Physiological circadian rhythms, normal social interaction, short preoperative fasting, frequent visits from family and friends after surgery, functional behavioral exercises for postoperative patients, humanistic care of family members, and early discharge from the hospital may contribute to lower postoperative cognition impairment.^33^, ^34^ Among various interventions, early prevention and early diagnosis are very vital to prevent and reduce postoperative cognitive decline.

In a word, the occurrence and development of POCD is the result of a combination of multiple factors in the perioperative period, and its etiology is multifactorial.^8^ There are no imaging or laboratory tests, patients therefore can only be diagnosed based on clinical observations. The exact mechanism of POCD has not been elucidated, resulting in a relative lack of effective drugs specific for the treatment of POCD, in addition to symptomatic and supportive care.^30^ The best treatment for POCD is unknown, and prevention appears to be the best treatment.

### Implications

How applicable are our estimates in clinical practice? Many studies^10^, ^35^, ^36^ suggest that increasing age and the education level are the specific risk factors of POCD, but they are difficult to be intervened. Other common independent risk factors for POCD include the 6‐minute walking distance, MMSE score, preoperative comorbidities, duration of surgery, intensive care unit length of stay, and postoperative complications.^10^, ^37^, ^38^ However, the available predictors reported for each trial varied. Finding sensitive and valid POCD predictors to accurately predict POCD risk remains a challenge. New rigorous research is also being explored.^39^, ^40^ Therefore, the current treatment strategies for these patients are multifaceted and this study may initially provide a valuable new approach to the prevention/early treatment of POCD from a new direction to facilitate early recovery.

### Limitations

Our study had at least seven limitations. First, this was a single‐center study with small sample size, and the sample size was not predetermined before the study. Thus, the association of intraoperative cerebral desaturation with POCD after spine surgery may have been underestimated. Larger multicenter studies are needed to confirm these associations. Second, this was an observational study, and therefore, somatic desaturation might be a manifestation or consequence of POCD rather than a causal mediator. Third, different NIRS monitors are commercially available, which allow either cerebral or peripheral tissue oxygenation monitoring. Differences in methodological approaches may lead to bias and variability between different devices.^41^ Therefore, study results obtained with different NIRS devices may not be applicable or comparable to each other, which might partially explain the conflicting results from published studies. Fourth, although the patients with hypertension included in this study were usually elderly (64.8 ± 9.3), its results did not apply to the elderly patients or all spinal surgery patients. What is more, elderly individuals generally have low education levels for historical reasons. Since education level may have a considerable influence on MMSE scores, the preoperative MMSE score in this study was relatively low. To ensure the representativeness of the study, we excluded patients with preoperative MMSE scores <18 rather than preoperative MMSE scores <24 (Fig. 1). Fifth, Only the MMSE was used for cognitive evaluation instead of using a validated battery of neuropsychological tests, which may highlight the limitations of the MMSE scale itself and may underestimate the incidence of POCD. Furthermore, the long‐term effects of POCD in this population were unclear due to the lack of later cognitive follow‐up assessments. Sixth, POCD in this study may include postoperative delirium on postoperative day 4 because it is not readily distinguishable in the early postoperative period. Seventh, other studies also used NIRS devices, and therefore, not all eligible patients were included, which may have increased the selection bias of the study.

### Conclusion and Outlook

In summary, the relatively high prevalence (27.7%) of POCD can occur in patients with hypertension undergoing open posterior spine surgery. Intraoperative SstO2 is associated with POCD after surgery, while SctO2 shows no association with POCD in this population. This study may initially provide a valuable new approach to the prevention/early treatment of POCD from a new direction. Therefore, conventional SctO2 monitoring might not allow a complete assessment of mismatches between oxygen supply and consumption during prone surgery. During prone surgery, the best monitoring locations, sensitive monitoring indicators, and their relationship to clinical outcomes require further clarification, especially in high‐risk patients undergoing high‐risk noncardiac surgery for whom few studies have been conducted.

## Author Contributions

The conception of the work: Fei Guo, Yeying Zheng, and Shuaiying Jia Acquisition, analysis, or interpretation of data for the work: Qiyan Wang, Wenzhang Wang, Qinyu Liu, Mingquan Hu, Qinyu Liu, and Shuaiying Jia. Drafting the work or revising it critically for important intellectual content: Fei Guo, Bin Lu, and Yeying Zheng All authors approved the final version of the manuscript, agree to be accountable for all aspects of the work in ensuring that questions related to the accuracy or integrity of any part of the work are appropriately investigated and resolved, and confirm that all persons designated as authors qualify for authorship, and all those who qualify for authorship are listed. Yeying Zheng is responsible for the overall content as guarantor.

## Supporting information


**Table S1.** Factors associated with the POCD: results from the univariable and multivariable logistic regression analysis.
