# Supplementary material for: Which Predictor, SctO2 or SstO2, Is more Sensitive for Postoperative Cognitive Dysfunction in Spine Surgery: A Prospective Observational Study?
Source: Orthop Surg. 2022 Nov 16;15(1):276–85. doi: 10.1111/os.13580 (PMC9837245; doi:10.1111/os.13580)
Supplement: Supplementary file 1 — Table S1. Factors associated with the POCD: results from the univariable and multivariable logistic regression analysis. [file OS-15-276-s001.doc]

**Supplementary Table S1. Factors associated with the POCD: results from the univariable and multivariable logistic regression analysis**

| **Variables** | **Univariable** | | |  | **Multivariable** | | |
| --- | --- | --- | --- | --- | --- | --- | --- |
|  | **OR** | **95%CI** | **P-values** |  | **OR** | **95%CI** | **P-values** |
| ASA III (vs. ASA II) | 3.393 | (1.348,8.538) | 0.009 |  | 3.992 | (1.434,11.112) | 0.008 |
| Platelet, per 10^9^/L | 1.012 | (1.004,1.021) | 0.005 |  | 1.012 | (1.003,1.021) | 0.009 |
| Postoperative sepsis | 3.750 | (0.928,15.160) | 0.064 |  | 5.201 | (1.057,25.583) | 0.042 |
| Age, per year | 1.052 | (1.000,1.106) | 0.049 |  | - | - | - |
| Education, per year | 0.921 | (0.825,1.029) | 0.146 |  | - | - | - |
| Chronic kidney disease | 3.750 | (0.928,15.160) | 0.064 |  | - | - | - |
| Decompression | 2.588 | (0.803,8.344) | 0.111 |  | - | - | - |
| Phenylephrine | 3.131 | (1.147,8.549) | 0.026 |  | - | - | - |
| Cerebrospinal leak | 5.072 | (1.124,22.890) | 0.035 |  | - | - | - |
| Postoperative 24h drainage volume, per ml | 1.004 | (1.000,1.007) | 0.056 |  | - | - | - |
| NPS in postoperative 3d, per point | 1.491 | (0.975,2.281) | 0.065 |  | - | - | - |

OR, odds ratio; CI, confidence interval; ASA, American Society of Anesthesiologists; NPS, numerical pain score
